# Impact of modulation of the endocannabinoid system on the intestinal microcirculation in experimental sepsis

**DOI:** 10.1186/cc9675

**Published:** 2011-03-11

**Authors:** C Lehmann, R Kuschnereit, I Kiister, J Zhou, S Whynot, O Hung, R Shukla, D Henzler, V Cerny, D Pavlovic, M Kelly

**Affiliations:** 1 Dalhousie University, Halifax, Canada

## Introduction

The endocannabinoid system (ECS) is upregulated during sepsis [1]. However, the functional outcomes of modulating endocannabinoid signaling during sepsis are currently unclear. Impairment of the intestinal microcirculation during sepsis may cause a breakdown of gut epithelial barrier function and bacterial translocation into the systemic circulation, increasing the systemic inflammatory response [2]. The aim of the present study was to examine the effects of CB1 and CB2 receptor modulation on the intestinal microcirculation in a model of polybacterial sepsis (colon ascendens stent peritonitis (CASP)) using intravital microscopy (IVM).

## Methods

We studied six groups of animals (Lewis rats, *n *= 10 per group): sham operated controls (SHAM), septic controls (CASP), CASP animals treated with CB1 agonist ACEA (2.5 mg/kg i.v.), CASP animals treated with CB1 antagonist AM281 (2.5 mg/kg i.v.), CASP animals treated with CB2 agonist HU308 (2.5 mg/kg i.v.), and CASP animals treated with CB2 antagonist AM630 (2.5 mg/kg i.v.). All treatments were performed immediately after sepsis induction. IVM of the intestinal microcirculation was performed 16 hours following sepsis induction. Leukocyte adhesion and functional capillary density were measured in a blinded fashion.

## Results

Following 16 hours of CASP-induced experimental sepsis, a significant increase of leukocyte adhesion in the intestinal submucosal venules (for example, collecting venules (V1): SHAM 35.7 ± 6.2 *n*/mm^2^, CASP 214.4 ± 22.6 *n*/mm^2^, *P *< 0.05) was observed. Capillary perfusion of the muscular and mucosal layers of the intestinal wall was significantly reduced (for example, longitudinalis muscular layer: SHAM 143.5 ± 7.6 cm/cm^2^, CASP 77.1 ± 7.2 cm/cm^2^). Treatment of CASP animals with the CB1 receptor agonist ACEA reduced leukocyte adhesion (V1 venules: 107.4 ± 5.1 *n*/mm^2^), whereas CB2 receptor stimulation did not affect leukocyte adhesion. However, CB2 receptor inhibition by AM630 reduced leukocyte activation significantly (V1 venules: 60.0 ± 14.1 *n*/mm^2^) and restored capillary perfusion (longitudinal muscular layer: 114.1 ± 7.6 cm/cm^2^).

## Conclusions

The data suggest that ECS signaling is involved in the impairment of the intestinal microcirculation during sepsis. Blocking CB2 receptor signaling reduces leukocyte activation and improves capillary perfusion in sepsis in rats. The long-term effect of ECS modulation needs further investigation.
